# Correlates of cervical cancer screening participation, intention and self-efficacy among Muslim women in southern Ghana

**DOI:** 10.1186/s12905-022-01803-0

**Published:** 2022-06-13

**Authors:** Nancy Innocentia Ebu Enyan, Anita Efua Davies, Rita Opoku-Danso, Francis Annor, Dorcas Obiri-Yeboah

**Affiliations:** 1 Department of Adult Health, School of Nursing and Midwifery, University of Cape Coast, Cape Coast, Ghana; 2 Directorate of Research, Innovation and Consultancy, University of Cape Coast, Cape Coast, Ghana; 3 Department of Microbiology and Immunology, School of Medical Sciences, University of Cape Coast, Cape Coast, Ghana

**Keywords:** Cervical cancer screening, Ghana, Intention, Modesty, Muslim women, Self-efficacy

## Abstract

**Background:**

The World Health Organisation’s efforts to eliminate cervical cancer by 2030 with a target of 70% screening coverage using a high-performance test demand that women increase participation in screening. Factors that impact uptake of screening must therefore be identified and bottlenecks addressed, especially in lower- and middle-income countries where cervical cancer incidence remains high. This study investigated Muslim women, participation in, intention to engage in and self-efficacy about cervical cancer screening.

**Methods:**

An analytical cross-sectional study was conducted among Muslim women aged 18 years and above in the Cape Coast Metropolis of Ghana using an interviewer-administered questionnaire. Data were analysed using appropriate descriptive statistics, Chi-square test, point biserial correlation and binary logistic regression analysis.

**Results:**

The mean age of participants was approximately 31 years (*M* = 30.9, *SD* = 10.4). Out of the 431 women, 21 (4.9%) had ever participated in cervical cancer screening. Participants demonstrated very low knowledge about cervical cancer and screening, with a mean knowledge score of 3.68 out of 15. Knowledge about cervical cancer was associated with increased odds of participating in cervical cancer screening (aOR = 1.32, 95%CI 1.11, 1.56). Concerns about similarity with health provider in terms of gender and faith was associated with decreased odds of cervical cancer screening self-efficacy (aOR = 0.81, 95% CI 0.67). Islamic modesty (aOR = 0.88, 95%CI 0.81, 0.96) was associated with decreased self-efficacy about seeking cervical cancer screening, whereas attitude (aOR = 1.32, 95%CI 1.14, 1.53) was significantly associated with increased self-efficacy about seeking cervical cancer screening. Again, Islamic modesty (aOR = 0.88, 95%CI 0.80, 0.97) was associated with decreased intention to participate in screening, whereas attitude (aOR = 1.42, 95%CI 1.20, 1.68) was associated with increased intention to participate in screening.

**Conclusions:**

There are gaps in knowledge of cervical cancer among Muslim women in this study as less than 5% had participated in screening. A positive attitude was found to influence intention to screen and actual participation in screening programmes. Islamic modesty and commitment to the Islamic faith decreased intention and self-efficacy regarding screening. Therefore, comprehensive and appropriate socio-cultural and religion-specific interventions aimed at addressing the barriers to screening are important in improving uptake among Muslim women.

## Background

Cervical cancer is a disease of global health concern. Over 570,000 women were diagnosed with the disease in 2018 worldwide and approximately 311,000 died from it [1]. The disease disproportionately affects women in low-and middle-income countries (LMIC) as 90% of all death occurred in those regions in 2018 [1]. Although there has been some improvement in high-income settings, the disease remains an important public health threat to women in resource-constraint settings [2]. This could be due to the unavailability of technologies in most health facilities in LMIC to detect the disease early, lack of available screening, inadequate information about the disease and lack of effective policies and guidelines about screening [2, 3]. Additionally, lack of treatment and vaccines are key drivers of cervical cancer morbidity and mortality in LMICs [1].

All sexually active women are at risk of infection with the Human Papillomavirus (HPV) and, therefore, tend to develop cervical cancer [4]. It is well documented that polygamous relationships and multiple sexual partners are some of the risk markers for contracting the virus [5]. In Ghana, polygamy is said to be practised [6] and is mostly seen in Muslim communities. Agboklu asserted that Islamic marriages are potentially polygamous [7]; thus, Muslim women may have higher chances of being exposed to HPV. However, Muslim women may have challenges with participating in cervical cancer screening (CCS) activities due to religious and cultural values [8].

Cervical cancer prevention strategies require early detection and management. A previous study conducted among American Muslim women revealed that delays in accessing health services were due to a perceived lack of female health care providers [9]. Concerns about modesty have been found to negatively impact the utilisation of preventive health screening by Muslim women, as they are less likely to participate in discussions on HPV due to feelings of embarrassment and fear [10]. Unpublished data from the health service indicates that females form just about 10% of the total number of obstetrics and gynaecology specialists in the country.

Inadequate information about Muslim women regarding cervical cancer and screening may lay a foundation for neglect in decision making and lack of cultural understanding and competent care regarding cervical cancer screening. This study investigated Muslim women’s participation in, intention to engage in, and self-efficacy about CCS.

## Methods

### Study design and participants

**Fig. 1:**
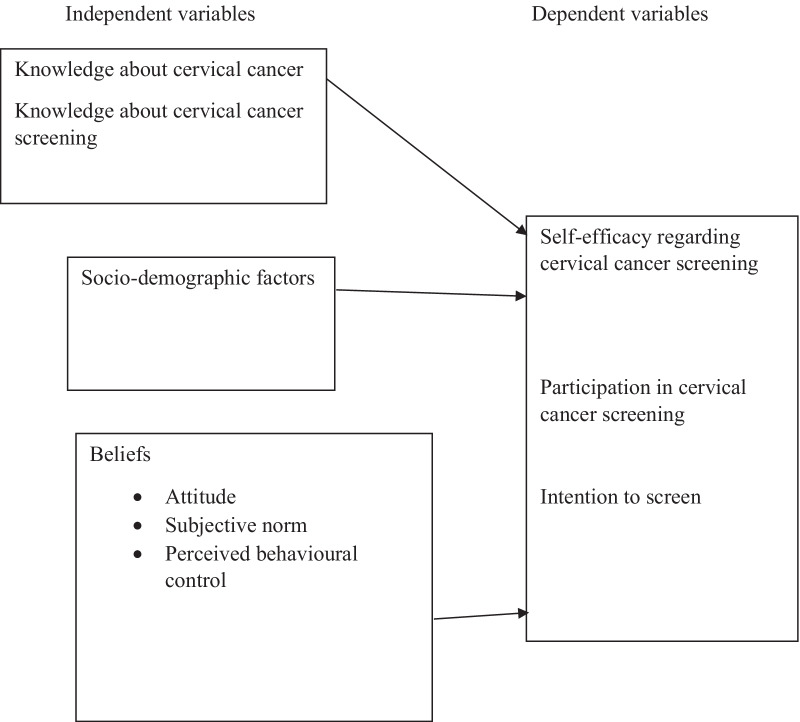
Conceptual framework of the study. *Source*: Adapted from Theory of Planned Behaviour [11] and Health Belief Model [12]

This study employed an analytical cross-sectional survey design. Figure 1 shows that the study was conceptualised within the Theory of Planned Behaviour (TPB) and the Health Belief Model (HBM), as they explain and predict health behaviours. To determine the CCS behaviours of Muslim women, the study adopted attitude, subjective norms and perceived behavioural control from the TPB. Attitude in this theory refers to the extent to which an individual has a favourable or unfavourable evaluation regarding the behaviour of interest. The subjective norm describes the individual’s beliefs about whether friends/peers and significant others approve or disapprove of the behaviour. Perceived behavioural control describes the individual’s perception of the facilitators and barriers to performing the behaviour while intention refers to the factors that motivate an individual to engage in a given behaviour in which stronger intention increases the likelihood of a behaviour being performed. In this study, attitudes, subjective norms, and perceived behavioural control were assessed in relation to CCS.

The concepts considered from the HBM were self-efficacy, knowledge and socio-demographic variables. Self-efficacy highlights the person’s level of confidence in engaging in the behaviour. Knowledge is a structural variable, which along with socio-demographics, is expected to enhance understanding of the issues regarding Muslim women’s CCS behaviours.

The study was conducted in the Cape Coast Metropolis located in the southern part of Ghana. Muslim women aged 18 to 66 years participated in the study. The Cape Coast Metropolis is estimated to have a female population of 87,084, of which 9.8% (8534) are Muslims [13]. Sexually active Muslim females 18 years and above who consented to participate in the study were included. Although screening is recommended for women aged 30 years and above in Ghana [14], this study included 18-year-olds to obtain information regarding cervical cancer and screening so that targeted education can be offered to enable them to engage in appropriate behaviours to prevent the disease. It is worth mentioning that for women living with HIV, it is recommended that they begin screening at the age of 25 years [14].

Considering the size of the population, the aim of the study, level of precision ± 5%, the confidence level of 95% and degree of variability of 50%, a minimum sample size of 382 was estimated for the study, using a formula suggested by Glenn [15]. First, four communities—Abura, Kotokuraba, Ola, and the University of Cape Coast—were randomly selected from the Cape Coast Metropolis using the simple random sampling with replacement technique. Second, the probability proportionate to size method was used to determine the number of Muslim women to participate in the study from each community. Third, a systematic sampling method was used in selecting the households in the varied communities. All eligible women at the household level who were willing to participate and consented were included in the study. In all, 439 women were invited to participate in the study, of which 431 completed the study, representing a response rate of approximately 98.2%.

### Data collection

The data collection for this study took place from February to April 2021 and involved the use of an interviewer-administered questionnaire. Five professional nurses who understand and communicate effectively in Fante and English were recruited and trained to collect the data. The training covered how the questionnaire should be administered and things to avoid in order not to introduce errors into the data. It took approximately 15 to 20 min to administer each questionnaire. Coronavirus disease (COVID-19) protocols were all adhered to during data collection. The data collection exercise took place at a convenient place in the homes of the participants where they were fully assured of privacy. Gatekeepers including Muslim leaders and volunteers assisted in identifying the households. The women were given education on the condition and directed to where they can access screening and more information after their knowledge about the disease had been assessed.

Before data collection, the survey instruments were presented to two experts to review the items in relation to the research questions. A pre-test was conducted with 50 Muslim women in Elmina Zongo. The reliability of the multi-item scales was assessed using Cronbach’s alpha or Kuder-Richardson (KR) 20, where appropriate. The results of the pre-test suggested that the survey instruments were found to be reliable and appropriate for the study.

## Measures

The instrument for data collection was a questionnaire. Most of the items were adapted from Ebu et al. [3] and Walton et al. [16] with a few new items developed based on existing literature.

Knowledge of cervical cancer was measured with 15 items covering the definition, risk factors, signs and symptoms and screening. Each item had a response option of “No” and “Yes”. Responses were scored as either correct (1) or wrong (0). A composite score was obtained for each participant by summing the number of correct responses. The internal consistency reliability estimate for this measure in this study is 0.89.

Participation in cervical cancer screening: Participants were asked to indicate “Yes (1)” or “No” (0) to whether they had ever been screened for cervical cancer. Participants were also asked to indicate whether they agreed or disagreed with 10 additional statements on their reasons for engaging in CCS. Those who had ever engaged in CCS were asked whether they would like to be involved in the exercise in future, whereas those who had never done the screening previously were asked to indicate whether they would like to do it for the first time. Again, responses to these questions were “Yes” or “No”.

Religion-related factors: We assessed participants’ beliefs in Islamic modesty with six items (α = 0.75; e.g., “I cover the whole body, leaving only the hands and face when in public/outside the home”). Additional three items were used to measure the extent of participants’ beliefs in the Islamic faith (α = 0.81; e.g., I will never get cervical cancer because Allah is in control). Each item had four response options ranging from “Strongly disagree” (1) to “Strongly agree” (4).

Provider-related factors: Two items were used to measure the extent to which participants emphasized their similarity with the health provider in terms of gender and religion (e.g., The gender of the provider is not a concern to me). Participants were also asked to indicate the extent to which they prefer health providers that allow self-sampling. Responses were made on a four-point Liker scale ranging from “Strongly Disagree” (1) to “Strongly Agree” (4).

Theory of planned behaviour constructs: Attitudes were measured with five items (α = 0.59; e.g., “I don’t think cervical cancer screening can promote my health”). Behavioural control was measured with four items (α = 0.74; e.g., “There is no cervical cancer screening facility in my community). Subjective norms were measured with six items (α = 0.68; e.g., “My relatives will encourage me to screen for cervical abnormalities”). Each item was rated on a four-point scale from “Strongly Disagree” (1) to “Strongly Agree” (4).

Socio-demographic factors: We obtained data on participants’ demographic characteristics including age, marital status, availability of health insurance, level of education, and employment status (Table 1).

**Table 1:** Participants’ demographic information

Variables/categories	Frequency	Percent
*Marital status*		
Single	197	48.0
Married	146	35.6
Divorced	11	2.7
Widowed	21	5.1
Cohabiting	35	8.5
*Level of education*		
Primary	110	26.8
Junior High School	67	16.3
Senior High School	78	19.0
College degree	28	6.8
University degree	76	18.5
No formal education	51	12.4
*Employment status*		
Employed	167	40.7
Unemployed	150	36.6
Student	93	22.7
*Income level*	410	100
Less than GH¢366	210	51.2
Between GH¢ 366–500	75	18.3
Between GH¢ 501–700	48	11.7
Between GH¢ 701–900	34	8.3
GH¢905 and above	43	10.5
*Age*		
18–29	234	54.3
30–39	114	26.5
40–49	58	13.5
50 +	25	5.8

The main concepts used in this study have been operationally defined as follows: Attitude refers to Muslim women’s favourable or unfavourable evaluation regarding participation in CCS. Subjective norm describes Muslim women’s beliefs about friends or family and significant others approving or disapproving of their participation in CCS. Perceived behavioural control refers to Muslim women’s perception of the factors that will either facilitate or impede participation in CCS. Intention refers to the behavioural willingness or decision to obtain CCS in the near future or otherwise. Self-efficacy is the level of confidence Muslim women have in participating in CCS.

### Data management and analysis

The data were checked for completeness and entered into the Statistical Package for Social Sciences version 21.0 for cleaning and analysis. The data was password protected and access limited to the members of the research team. The data were analysed using frequency counts, percentages, mean, standard deviation, Chi-square test, point biserial correlation and binary logistic regression analysis. First, we conducted descriptive statistics on participants’ demographic characteristics, knowledge of cervical cancer, and participation in CCS. Next, using the chi-square test and point biserial correlations, we examined bivariate associations between demographic factors and participation in CCS, intention to engage in CCS, and self-efficacy about CCS. Finally, using binary logistic regression analysis, we examined the extent to which demographic factors, religion and modesty factors, provider-related factors, and theory of planned behaviour factors are associated with participation in CCS, intention to do CCS, and self-efficacy about CCS.

## Results

### Participants’ demographic information

As shown in Table 1, the participants were aged between 18 and 66 years with a mean age of approximately 31 years (*M* = 30.9, *SD* = 10.4), and the majority (52%) were not married. Participants had varied educational levels with a little of half having up to secondary education, one-fourths with a college/university degree, and a few with no education. In terms of earnings, the majority of the participants earned less than GH¢366.00 (USD60.00) a month. Close to half of the participants were employed while about a third were unemployed and the rest were students.

### Knowledge of cervical cancer and participation in cervical cancer screening

Participants responded to a series of questions meant to test their knowledge of cervical cancer and CCS. Table 2 presents details of participants’ responses to these questions. A sum of the correct responses across the 15 questions was computed for each participant. Overall, the participants showed poor knowledge of the nature of cervical cancer, its symptoms, common risk factors and screening. A majority (75%) of the participants answered less than half of the fifteen questions correctly (Table 2).

**Table 2:** Participants’ knowledge of cervical cancer and cervical cancer screening

Item	Incorrect response	Correct response
Cervical cancer affects the mouth	270 (62.6)	161 (37.4)
Cervical cancer affects the face	316 (73.3)	115 (26.7)
Cervical cancer cannot be prevented	336 (78.0)	95 (22.0)
Being transfused with blood is a risk factor for CC	363 (84.2)	68 (15.8)
Having multiple sexual partners increase your CC risk	278 (64.5)	153 (35.5)
CC is heredity	377 (87.5)	54 (12.5)
Bleeding after sexual intercourse is a CC symptom	303 (70.3)	128 (29.7)
Offensive blood-stained vaginal discharge is a CC symptom	328 (76.1)	103 (23.9)
Itching around the vulva is a CC symptom	390 (90.5)	41 (9.5)
Severe headache all the time is a CC symptom	384 (89.1)	47 (10.9)
Screening can prevent cervical cancer	253 (58.7)	178 (41.3)
Screening can cure cervical cancer	382 (88.6)	49 (11.4)
Urine is used for cervical screening in Ghana	375 (87.0)	56 (13.1)
CC Screening must be done by a trained person	232 (53.8)	199 (46.2)
CC Screening cannot be obtained in Ghana	293 (68.0)	138 (32.0)
*Summary statistics*		
Minimum score = 0		
Maximum score = 14.0		
Mean score = 3.68		
Standard deviation = 3.88		
75% percentile = 7.00		

As shown in Table 3, participation in CCS exercise was considerably low among the research participants. Only less than 5% of the participants had engaged in CCS. Participants reported multiple reasons for their engagement in CCS. In most cases (90%), participants engaged in CCS because they received education about cervical cancer at a health facility. In many other instances (70%), participants engaged in CCS because they heard about it on the radio. In some cases, participants engaged in CCS because they had experienced some symptoms; were referred or requested to do the test; fear cervical cancer, or were involved in a routine check-up. Encouragements from husbands, having a relative with cervical cancer, and education on cervical cancer at the mosque were the least mentioned reasons for participants’ engagement in CCS. Most of the participants (80%) who had engaged in CCS expressed no intention of engaging in the screening exercise again.

**Table 3:** Participation and reasons for cervical cancer screening

	Yes *n* (%)	No *n* (%)
Ever had cervical cancer screening (*n* = 431)	21 (4.9)	410 (95.1)
Reason for cervical cancer screening (*n* = 20)		
Routine	6 (30.0)	14 (70.0)
Referral	10 (50.0)	10 (50.0)
I asked for the test	10 (50.0)	10 (50.0)
Husband encouraged me	4 (20.0)	16 (80.0)
A relative had cervical cancer	4 (20.0)	16 (80.0)
Fear of cancer	7 (35.0)	13 (65.0)
Heard about it on the radio	14 (70.0)	6 (30.0)
Experienced some symptoms	10 (50.0)	10 (50.0)
I had education on cervical cancer at the mosque	5 (25.0)	15 (75.0)
I had education on cervical cancer at the hospital	18 (90.0)	2 (10.0)
Intend to have cervical cancer screening again (*n* = 20)	4 (20.0)	16 (80.0)

### Bivariate associations

We performed cross-tabulations with chi-square tests to examine bivariate associations between categorical demographic variables and the three main outcome variables in the study (participation in CCS, intention to engage in CCS, and self-efficacy about CCS). As shown in Table 4, monthly income (χ^2^ (2) = 14.87, *p* < 0.01) and educational level (χ^2^ (2) = 15.81, *p* < 0.01) were significantly associated with participation in CCS. Women with below-average monthly incomes were less likely to engage in CCS than those with average and above-average incomes. Likewise, women with at least secondary level education were more likely to engage in CCS than those with lower levels of education. Subscription to health insurance (χ^2^ (1) = 5.18, *p* < 0.05) was the only demographic variable that showed significant bivariate association with intention to engage in CCS. Women with health insurance were more likely to express intention to engage in CCS than those without health insurance. Marital status (χ^2^ (1) = 3.89, *p* < 0.05), health insurance (χ^2^ (1) = 6.55, *p* < 0.05), and employment status (χ^2^ (1) = 8.88, *p* < 0.05) had significant bivariate associations with self-efficacy about CCS. Women with partners (married or co-habiting) and those with health insurance were more likely to express self-efficacy about CCS, whereas students were less likely to express self-efficacy about CCS. Using point biserial correlation, we found that age was not significantly associated with any of the outcome variables.

**Table 4:** Bivariate associations between demographic variables and outcome variables

Variable	Participated in CCS (*n* = 431)			CCS intention (*n* = 410)			CCS self-efficacy (*n* = 431)		
	No	Yes	χ^2^	No	Yes	χ^2^	No	Yes	χ^2^
	n (%)	n (%)		n (%)	n (%)		n (%)	n (%)	
Marital status			1.37			0			3.89*
Single	229 (96.2)	9 (3.8)		34 (14.8)	195 (85.2)		49 (20.9)	185 (79.1)	
With partner	181 (93.8)	12 (6.2)		27 (14.9)	154 (85.1)		26 (13.6)	165 (86.4)	
Health insurance			0.78			5.18*			6.55*
No	51 (52.3)	4 (7.3)		13 (25.5)	38 (74.5)		16 (30.2)	37 (69.8)	
Yes	359 (95.5)	17 (4.5)		48 (13.4)	311 (86.6)		59 (15.9)	313 (84.1)	
Employment status			3.77			0.65			8.88*
Employed	167 (94.9)	9 (5.1)		22 (13.2)	145 (86.8)		23 (13.1)	152 (86.9)	
Unemployed	150 (97.4)	4 (2.6)		24 (16.0)	126 (84.0)		25 (16.6)	126 (83.4)	
Student	93 (92.1)	8 (4.9)		15 (16.1)	78 (83.9)		27 (27.3)	72 (72.7)	
Monthly income			14.87**			3.74			4.69
Below-average	210 (99.1)	2 (0.9)		35 (16.7)	175 (83.3)		39 (18.6)	171 (81.4)	
Average	123 (92.5)	10 (7.5)		12 (9.8)	111 (90.2)		16 (12.3)	114 (87.7)	
Above-average	77 (89.5)	9 (10.5)		14 (18.2)	63 (81.8)		20 (23.5)	65 (76.5)	
Education level			15.81**			1.67			1.51
Up to primary	228 (98.3)	4 (1.7)		37 (16.2)	191 (83.8)		36 (15.7)	193 (84.3)	
Secondary	78 (84.7)	11 (12.4)		8 (10.3)	70 (89.7)		16 (18.4)	71 (81.6)	
Degree	104 (94.5)	6 (5.5)		16 (15.4)	88 (84.6)		23 (21.1)	86 (78.9)	

*p < 0.05; **p < 0.01

### Multivariate associations

Three separate binary logistic regression analyses were conducted to examine multivariate correlates of participation in CCS, self-efficacy about CCS, and intention to engage in CCS for the first time. As shown in Table 5, the final models for participation in CCS (χ^2^ (12) = 51.72, *p* < 0.001), CCS intention (χ^2^ (12) = 50.11, *p* < 0.001), and CCS self-efficacy (χ^2^ (12) = 54.33, *p* < 0.001) were statistically significant. Having an average (aOR = 5.87, 95% CI 1.07, 32.27) or an above-average (aOR = 7.95, 95% CI 1.36, 46.28) monthly income was associated with greater odds of engaging in CCS than having below-average income. Knowledge about cervical cancer was associated with increased odds of engaging in CCS (aOR = 1.32, 95% CI 1.11, 1.56). None of the religion/modesty and provider-related factors was significantly associated with the odds of engaging in CCS.

**Table 5:** Binary logistic regression on correlates of engagement in cervical cancer screening, self-efficacy about cervical cancer screening and intention to engage in cervical cancer screening

Variables	Participated in CCS (*n* = 431)			CCS self-efficacy (*n* = 431)			CCS intention (*n* = 410)		
	*B* (S.E.)	aOR	95% CI	*B* (S.E.)	aOR	95% CI	*B* (S.E.)	aOR	95% CI
*Demographics*									
Monthly income^a^									
Average	1.77 (0.87)*	5.87	1.07–32.27	0.01 (0.37)	1.01	0.49–2.10	0.15 (0.42)	1.16	0.51–2.63
Above-average	2.07 (0.90)*	7.95	1.36–46.28	− 0.41 (0.38)	0.66	0.32–1.39	− 0.38 (0.43)	0.68	0.29–1.59
Education level^a^									
Secondary	1.02 (0.71)	2.76	0.68–11.20	− 0.16 (0.38)	0.85	0.40–1.81	0.76 (0.47)	2.13	0.85–5.38
Degree	− 0.64 (0.86)	0.53	0.99–2.83	− 0.47 (0.42)	0.62	0.28–1.41	0.45 (0.47)	1.56	0.62–3.95
*Religion factors*									
Modesty	0.22 (0.07)	1.24	1.07–1.43	− 0.13 (0.04)***	0.88	0.81–0.96	− 0.13 (0.05)***	0.88	0.80–0.97
Faith	0.01 (0.13)	1.01	0.79–1.29	− 0.00 (0.79)	0.99	0.86 –1.17	− 0.05 (0.09)	1.05	0.88–1.26
*Provider-related*									
Self-sampling	0.18 (0.78)	1.20	0.26–5.52	− 0.24 (0.42)	0.79	0.35–1.79	− 0.92 (0.50)	0.40	0.15–1.06
Similarity	0.11 (0.17)	1.11	0.80–1.55	− 0.21 (0.10)*	0.81	0.67–0.99	− 0.18 (0.11)	0.84	0.67–1.04
*TPB variables*									
Knowledge	0.28 (0.09)**	1.32	1.11–1.56	0.06 (0.05)	1.06	0.97–1.17	− 0.03 (0.05)	0.97	0.88–1.08
Attitudes	0.24 (0.14)	1.28	0.98–1.67	0.28 (0.08)***	1.32	1.14–1.53	0.35 (0.09)***	1.42	1.20–1.68
Subjective Norms	− 0.10 (0.08)	0.91	0.77–1.06	− 0.02 (0.05)	0.98	0.89–1.07	0.00 (0.06)	1.00	0.90–1.11
Behavioural Control	0.07 (0.12)	1.07	0.85–1.36	0.03 (0.06)	1.03	0.92–1.16	0.09 (0.06)	1.10	0.97–1.24

*TPB* Theory of planned behaviour, *aOR* Adjusted odds ratio, *CCS* Cervical cancer screening, *CI* Confidence interval

^a^Reference group (monthly income = below-average; education level = up to primary education)

*p < 0.05; **p < 0.01; ***p < 0.001

No demographic variable was shown to have a statistically significant association with CCS intention and self-efficacy about CCS in the final logistic regression models. Concerns about the similarity with health providers in terms of gender and faith were associated with decreased odds of CCS self-efficacy (aOR = 0.81, 95% CI 0.67, 0.99). Islamic modesty (aOR = 0.88, 95% CI 0.81, 0.96) was associated with decreased self-efficacy about CCS, whereas attitude (aOR = 1.32, 95% CI 1.14, 1.53) was significantly associated with increased self-efficacy about seeking CCS. Again, Islamic modesty (OR = 0.88, 95% CI 0.80, 0.97) was associated with decreased intention to engage in CCS, whereas attitude (aOR = 1.42, 95% CI 1.20, 1.68) was associated with increased intention to engage in CCS.^1^

## Discussion

### Knowledge of cervical cancer and participation in screening

The study examined Muslim women’s knowledge of cervical cancer, CCS behaviour, and reasons for screening. It also investigated the correlates of participation in CCS, self-efficacy about CCS, and intention to engage in CCS for the first time. Our findings highlight gaps in knowledge of cervical cancer and CCS. For instance, more than two-thirds of the participants incorrectly indicated that cervical cancer affects the face and specified that it cannot be prevented. Studies conducted in Africa and the United Arab Emirates have reported similar findings [17–19]. Our study further found knowledge about cervical cancer to be associated with increased odds of engaging in CCS. Other studies conducted within the African context reported similar findings [20, 21]. Therefore, education on cervical cancer highlighting the risk factors, signs and symptoms and methods of prevention could lead to improved screening uptake. It is worth mentioning that an interventional study conducted in Ghana reported that targeted health education on cervical cancer and screening had an impact on changing the perceptions of women regarding the disease and screening in a district in Ghana [22].

Surprisingly, regarding CCS, above 80% of the participants indicated that urine was used in CCS and screening can prevent cervical cancer, respectively. Although previous studies have reported that high-risk HPV types can be detected in urine samples [23, 24], it is not recommended by WHO and hence not a routine practice. Additionally, urine is not an option in the Ghanaian setting and based on the population studied, it is most likely the case that they chose it based on the fact that most gynaecological and obstetric investigations they have experienced involved using a urine sample.

Participation in CCS was extremely low, as less than 5% of the women studied had ever had in CCS. Similarly, women attending maternal and child health clinics in Ethiopia reported low knowledge of CCS [25]. The low level of public awareness about CCS and inadequate screening facilities in hospitals across Ghana could account for the observed finding. It was observed in Ontario that Muslim women of sub-Saharan African (SSA) origin had the highest prevalence of being behind schedule regarding CCS compared to those from non-Muslim SSA backgrounds. This suggests that Muslim women from SSA settings have a decreased likelihood of being informed about CCS as utilisation of primary health care facilities has effects on the uptake of screening services [26]. The fundamental gaps in information about screening cannot be ruled out despite the differences in settings with the current study. In contrast, Muslim women in Monrovia showed much interest in screening by stating their preference for self-sampling [16] which highlights the need for community engagement with religious leaders in eliciting and incorporating culturally appropriate identified strategies to encourage participation.

Furthermore, women in this study cited reasons for engaging in CCS, which include education on CCS at the hospital, information on the radio, referral, experiencing some symptoms and requesting the test. Pragmatic and intentional efforts to improve screening uptake need to combine these strategies. For instance, studies conducted in Nigeria and Iran found referral by doctors and other healthcare providers to improve Pap testing uptake [27, 28]. Efforts to intensify education on CCS in health facilities and engagement of the mass media could be effective in assisting women with the needed information to make appropriate decisions about screening. This would help prevent late diagnosis and reduce mortality from cervical cancer. Given that cervical cancer is usually symptomless in its early stages, early screening can detect abnormal changes in the cervix before progressing to cancer.

### Correlates of participation in CCS, confidence about CCS, and intention to engage in CCS for the first time

The study found that having an average or an above-average monthly income was associated with an increased likelihood of engaging in CCS than having a below-average income. This finding was reported in a South African study involving a secondary data analysis of the Demographic and Health Survey data [29]. The National Health Insurance Scheme in Ghana presently does not cover cervical cancer screening. Participation in screening is based on the ability to pay at the point of accessing the service. Hence, women with below-average monthly incomes are less likely to participate in CCS activities as reported in this study. Couple with the issue of payment at the point of service use is limited screening facilities in Ghana that requires additional financial resources to enable women to travel to seek screening services.

Education predicted CCS in the current study. Women with a secondary level of education were more likely to engage in CCS than those with lower levels of education, while those with a college/university degree made no difference in the odds of engaging in CCS. This finding is consistent with studies conducted within the SSA setting and beyond [3, 30, 31]. Mitiku and Tefera reported that women with a secondary level of education were more likely to seek CCS compared to those without any formal education [31]. A possible explanation is that those with secondary education may have abided by the health education messages and doctors’ recommendations compared to those with lower levels of education as they may lack information about the disease and screening. Women with a university or college degree may have high health literacy and access to more educational materials about the disease and screening and therefore may be indifferent toward screening.

Furthermore, among the theory of planned behaviour constructs that guided this study, only attitude had a significant association with intention to screen and participation in CCS, with positive attitude being associated with increased odds of CCS. Attitude in this study refers to Muslim women’s favourable or unfavourable evaluation toward participating in CCS. Intention to participate in CCS depends on a person’s attitude since it is a strong determinant of intention [32] which is consistent with the current study. This implies that when such women are provided with the necessary resources and opportunities their attitude to screening could be modified to achieve a desirable outcome. Compared to the findings of the current study, all the TPB constructs influenced the intention to engage in CCS in an institution-based study conducted in Ethiopia in 2017 [32]. A similar result was achieved in a study among Chinese women [33]. The characteristics of the population used could account for the difference in findings. For instance, over 85% of the participants sampled were Christians of either the Protestant or Orthodox denomination while the current study solely focused on Muslims. Hence, the difference in faith might have contributed to the different study outcomes.

Religious faith and concerns about modesty were important in determining CCS participation, intention and self-efficacy. Although Muslim women are a minority group compared to the other religious groups, a lack of cultural understanding of their religious beliefs and values could be a critical barrier to seeking CCS. Islamic modesty was associated with decreased intention to engage in CCS in this study. Additionally, Islamic modesty and commitment to the Islamic faith were associated with decreased self-efficacy about seeking CCS. The findings are consistent with previous studies [10, 34, 35]. A survey conducted among a sample of Muslim women in Chicago to determine religion-related factors associated with Papanicolaou (Pap) testing found modesty levels to be negatively associated with Pap testing [34]. A possible explanation is the inherent beliefs of Muslim women, as they find it problematic for male doctors to examine their ‘private parts’ and others need approval from their partners before being examined by male doctors or participating in gynaecological examinations. Consistent with this finding is a study conducted among Nigerian women in which approximately half of the women were Muslims. The authors found spousal approval, religious and cultural commitment and the gender of the healthcare provider to be important barriers to seeking CCS [36]. Given the peculiar issues confronting Muslim women regarding CCS, self-collection of samples for HPV screening can play an important role to improve the acceptability of screening and uptake [19]. For instance, in Liberia, Muslim women showed their preference for self-sampling over the conventional provider collection [17]. This highlights the need for a tailor-made programme aimed at teaching and encouraging Muslim women to take advantage of the self-collection method to participate in CCS since its convenient and can be collected in their homes [37]. A narrative from key informants’ interviews conducted among Muslim women in New Yorke City indicates that Islam emphasizes the importance of personal health management which makes it imperative for Muslim women to participate in CCS programmes. However, sociocultural gender norms where the female is expected to place the health of family and significant others before her health may be a barrier [38]. Although the context differs from the current study, the findings are relevant as limited studies have been conducted in Africa among this population. An important limitation worth noting is that this study focused on only Muslim women. Therefore, the views of women belonging to other religious sects were not captured. It is suggested that future studies compare CCS behaviours of women belonging to different religious sects to understand their unique issues and implement strategies to deliver culturally-sensitive screening programmes.

## Conclusions

Our study highlights gaps in knowledge of cervical cancer and screening among Muslim women which call for comprehensive culturally-appropriate health education to improve knowledge and change attitudes toward CCS. A positive attitude was found to influence intention to screen and actual participation in screening programmes. The study identified that less than 5% had participated in CCS. Reasons cited for participation in CCS were education at the hospital, referral, information on the radio and experience of symptoms. Moreover, average monthly income and having secondary education increased the likelihood of seeking CCS. Interventions to improve the level of education of Muslim women and income are critical in facilitating CCS.

However, religion/modesty and provider-related factors decreased the likelihood of CCS. Islamic modesty and commitment to the Islamic faith decreased intention and self-efficacy regarding screening. Therefore, socio-cultural interventions aimed at tackling the religion/modesty and provider-related factors are important in improving CCS uptake among Muslim women.

## Data Availability

The datasets used and/or analysed during the current study are available from the corresponding author on reasonable request.

## Abbreviations

CC: Cervical cancer
CCS: Cervical cancer screening

## Footnotes

1 Based on the suggestion of an anonymous reviewer, we explored the possibility that the influence of knowledge on the outcome variables may depend on participants’ age. Although we found a marginal negative correlation between age and knowledge of cervical cancer (*r* = –.15, *p* < .05), results from separate logistic regression analyses indicated that the interaction between age and knowledge of cervical cancer had no significant influence on any of the outcome variables (participation in CCS, CCS intention, and CCS self-efficacy). Thus, the extent to which knowledge relates to participation in CCS, intention to screen, and self-efficacy about CCS was not influenced by age.
